# The Microscope and Microscopical Technology

**Published:** 1880-03

**Authors:** 


### BIBLIOGRAPHICAL.

---

THE MICROSCOPE AND MICROSCOPICAL TECHNOLOGY—A Text-Book for physicians and students. By Heinrich Frey, Prof. of Medicine in the University of Zurich. Translated and Edited by George R. Cutter, M. D., Surgeon New York Eye and Ear Infirmary etc., etc. Illustrated by three hundred and eighty-eight Engravings on wood—Second Edition—New York, Wm. Wood & Co., 1880.

To Microscopy we are indebted for many important practical facts connected with pathology, and hence works on this subject are almost indispensable to the practicing physician. The above work, an octavo volume of 660 pages placed on our table by Mr. Dalston, treats thoroughly of the subject. It gives the theory of the instrument and manner of testing it. Also its use and the preparation of objects preceded, by the description of normal and pathological tissues.

To the student and practitioner of microscopy this book will prove all that is desirable.

